# Menstrual cycle irregularity during examination among female medical students at King Abdulaziz University, Jeddah, Saudi Arabia

**DOI:** 10.1186/s12905-022-01952-2

**Published:** 2022-09-06

**Authors:** Maisam H. Alhammadi, Afaf M. Albogmi, Manar K. Alzahrani, Bashayer H. Shalabi, Fatma A. Fatta, Samera F. AlBasri

**Affiliations:** 1 Faculty of Medicine, King Abdulaziz University, Jeddah, Saudi Arabia; 2 Obstetrics and Gynecology Department, King Abdulaziz University, Jeddah, Saudi Arabia

**Keywords:** Menstrual irregularities, Dysmenorrhea, Exam, Medical student

## Abstract

**Background:**

Menstrual irregularity is defined as any differences in the frequency, irregularity of onset, duration of flow, or volume of blood from the regular menstrual cycle. It is an important medical issue that many medical students suffer from. The study aimed to determine the menstrual cycle abnormalities women experienced during exams and to investigate the most common types of irregularities among female medical students at King Abdulaziz University in Jeddah, Saudi Arabia.

**Methods:**

A cross-sectional study was conducted among female medical students between September and October 2021 at King Abdulaziz University in Jeddah, Saudi Arabia. For this study, the estimated sample size (n = 450) was derived from the online Raosoft sample size calculator. Thus, 450 female medical students from second to sixth year were selected through stratified random sampling. A validated online questionnaire collected data about demographics, menstrual irregularities during exams, type of irregularities, menstrual history, family history of menstrual irregularities, premenstrual symptoms, medication use, medical and family consultations, and absenteeism. The chi-squared test (χ^2^) was used to analyze the associations between variables.

**Results:**

A total of 48.2% of participants had menstrual irregularities during exams. The most common irregularity was dysmenorrhea (70.9%), followed by a lengthened cycle (45.6%), and heavy bleeding (41.9%). A total of 93% of medical students suffered from premenstrual symptoms and 60.4% used medication such as herbal medication and home remedies to relieve menstrual irregularities, and 12.1% of the students missed classes due to menstrual irregularities. A non-significant relationship was found between menstrual irregularities during exams and students’ demographics, academic year, and age at menarche, while oligomenorrhea, a heavier than normal bleed, a longer than normal cycle, and missing classes due to menstrual irregularities were significantly higher among single students as opposed to married students.

**Conclusion:**

The results showed that female medical students have a significant frequency of menstruation abnormalities during exams period. Colleges should raise awareness among medical students about coping with examination stress and seeking medical care for menstrual abnormalities.

## Introduction

The menstrual cycle is the most important measure of female reproductive health [1, 2]. It is a normal physiological process where the uterus eliminates the endometrium (innermost lining layer) and allows a regulated loss of endometrial tissue, mucus, and blood through the vagina every month [3]. Menstruation lasts 2 to 6 days and occurs between every 21 and 35 days (counting from the start of one cycle to the start of the next cycle), but this can vary from month to month depending on the woman [4].

Menstrual cycle irregularity is one of the most common reasons for women to see gynecologists and family physicians [5]. The menstrual cycle is considered to be irregular if there are any changes in frequency and irregularity of onset, duration of flow, and volume of blood from the regular menstrual cycle [6]. It can have significant implications for women's health and can lead to major health consequences, such as cardiovascular disease, infertility, Type 2 diabetes, and osteoporosis [7–12].

There are many risk factors in an irregular menstrual cycle. A study conducted in Korea on 4,788 women found that irregular menstruation is strongly associated with important factors, such as body mass index, perceived level of stress, and smoking status [13].Also, some evidence suggests that the risk of an irregular menstrual cycle tends to increase with mental health problems, such as anxiety and depression [14]. Another study in 2020 found that females who use nicotine are at a five-times higher risk of an irregular menstrual cycle. Other factors include marriage, having a cesarean section, and using contraception [5]. A 2019 study found that the prevalence of irregular menstruation is 11.83%; however, women with longer working hours have double the risk for irregular menstruation compared to women working shorter hours [11]. In recent years, nutrition and lifestyle have been linked to menstrual disorders. This has been primarily attributed to the effects of specific meals and practices on vascular irrigation or the amount of estrogens and prostaglandins—the hormones that regulate the menstrual cycle [15–19]. A study conducted in India found that irregular menstruation is the third most common menstrual disorder among Indian medical students, accounting for 29% of the total. An irregular menstrual cycle was most common among Lebanese nursing students (80.7%) [20]. A study completed in Dammam, Saudi Arabia, found that 91% of female students studying health sciences had menstrual disorders, including an irregular menstrual cycle (27%) [21]. A study in Jeddah, Saudi Arabia, among medical students at King Abdulaziz University, found that that 60.9% of students suffered from dysmenorrhea [22], and a study in Riyadh, Saudi Arabia, among female medical students in King Saud University found the prevalence of primary dysmenorrhea was 80.1% [23]. Menstrual issues have a detrimental influence on women's quality of life, resulting in a variety of constraints, absenteeism, and poor academic performance [24–26]. Although menstrual cycle irregularity is a major public health issue among females, little research has been undertaken on this critical issue among medical students in Jeddah. This study was conducted to identify menstrual cycle irregularities during exams among female medical students at King Abdulaziz University (KAU), Jeddah, Saudi Arabia.

## Study design and setting

A cross-sectional study was completed at KAU in Jeddah, Saudi Arabia between September and October 2021. KAU is a public university in Jeddah Which consider the largest city after Riyadh in Saudi Arabia, with student enrolment over 80,000. It is considered one of the top institutions of higher education in the Kingdom of Saudi Arabia [27], and has been ranked as the top Arab university by Times Higher Education [28].

### Study participants

Female medical students (n = 450) at KAU participated in the study. Medical education in KAU is about 6 years divided into two stages after freshman year. 2nd and 3rd year are basic sciences medicine. 4th, 5th, and 6th are clinical, and students learn by attending with physicians in a hospital setting. Participants were all female medical students, in their second to sixth years, including basic sciences and clinical medicine. The exclusion criteria included any female with chronic illness, gynecological diseases, or breastfeeding that might affect their period.

### Sample size determination

For this study, the estimated sample size was derived from the online Raosoft sample size calculator [29].The sample size was calculated based on a response rate of 50%, a confidence interval of 98%, and a margin of error of 5%, with a total population of about 1,390. Counting after freshman year, 2nd, 3rd, 4th, 5th, and 6th year female medical students at KAU, the largest required sample size is 400. Accordingly, this study included a randomized sample of 450 students.

### Data collection

Data collection was done using a pretested, structured and validated self-administered English questionnaire. After an evaluation of literature review, the questionnaire was changed and adjusted for the purpose of this study. The questionnaire was distributed between July to August 2021 among female medical students at KAU [30]. The questionnaire consisted of four parts. The first part included questions about socio-demographic data including age, marital status, academic year, weight and height, and age at menarche. The second part included questions about types of menstrual irregularities during exams. Amenorrhea was explained to students as absence of menstrual periods; oligomenorrhea was explained as infrequent menstrual periods (fewer than six to eight periods per year); and dysmenorrhea was explained as painful menstruation. Premenstrual symptoms were mentioned to students as mood swings, tender breasts, and food cravings. Questions were included on the type of medication used to relieve menstrual irregularities (allopathic, herbal medication, or home remedies) and whether the participant thought it was effective or not. The third part included questions about history of being diagnosed with chronic medical conditions such as (diabetes mellitus, hypertension, fibroids, polycystic ovary syndrome and others) and about family history of menstrual irregularities. The fourth part included questions about the impact of menstrual irregularities on the student such as missing classes during menstrual irregularities, seeking any medical help from a clinician, and discussing this problem with a family member or a friend.

The experts rated each item on a 4-point scale based on its suitability and relevance as follows: adequate (simple, relevant and clear) equal 4 point, adequate but needs minor revision equal 3 point, needs major modification equal 2 point ,or not so adequate (can be omitted) equal 1 point. The percentage of all items with a 3 or 4 rating from experts is known as the content validity index (CVI). With a 4-rating 80 percent of the time, a score is thought to have good validity. The CVI of the planned questionnaire was established. To assess the reliability, Cronbach's alpha value was determined as 0.82.

### Research ethics

This study was approved by King Abdulaziz University Hospital, biomedical ethical committee (Ref No. 384-21). All study participants were notified about the research objectives, and informed consent was obtained.

### Statistical analysis

Microsoft Excel 2016 was used for data entry, and SPSS version 26 was used to analyze the data. To assess the relationship between variables, qualitative data was expressed as numbers and percentages, and the chi-squared test (χ^2^) was used. Multivariate logistic regression analysis was done to assess the risk factors of menstrual irregularities. A *p*-value of 0.05 was considered statistically significant.

## Results

**Fig. 1:**
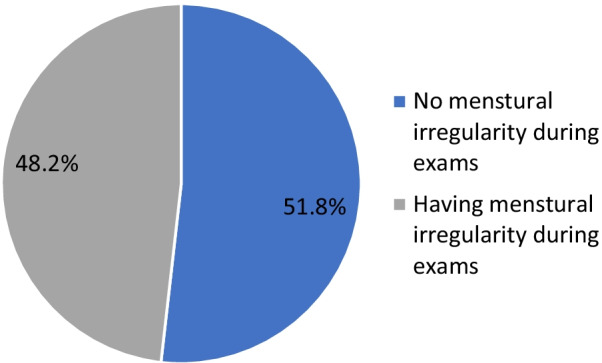
Percentage distribution of participants according to presence of menstrual irregularity during exams

Table 1 shows that 96% of studied students had an age ranging from 18 to 24 years; 98.2% were single, and 28.7% were fourth-year students. Figure 1 illustrates that 217 (48.2%) of participants had menstrual irregularities during exams.

**Table 1:** Distribution of participants according to their demographic data, academic year and age at menarche (N = 450)

Variable	No. (%)
*Age*	
18–24	432 (96)
25–31	18 (4)
*Marital status*	
Divorced	2 (0.4)
Married	6 (1.3)
Single	442 (98.2)
*Academic year*	
2nd	117 (26)
3rd	73 (16.2)
4th	129 (28.7)
5th	72 (16)
6th	59 (13.1)
Age at menarche (mean ± SD)	12.79 ± 1.55

Table 2 shows that the most common menstrual irregularity during exams was dysmenorrhea (70.9%), followed by normal cycle lengthened (45.6%), and heavier than normal bleed (41.9%). Of these, 93% suffered from premenstrual symptoms, and 60.4% used medication to relieve menstrual irregularities—both herbal medication and home remedies, and 82.4% reported the effectiveness of these. According to students' answers, we found that most of the participants have more than one type of menstrual irregularity. Family history of menstrual irregularities was present among 43.8% of students with menstrual irregularities during exams, and only 26.8% reported seeking medical help from a clinician. Of these, 76% discussed this problem with a family member or a friend, and 12.1% missed classes due to menstrual irregularities. A non-significant relationship was found between menstrual irregularities during exams and students’ demographics, academic year, or age at menarche (*p* > 0.05) (Table 3).

**Table 2:** Distribution of participants with menstrual irregularities during exams according to menstrual history (N = 217)

Variable	No. (%)
*Type of menstrual irregularities (N* = *217) (more than one answer was allowed)*	
Amenorrhea	86 (39.6)
Oligomenorrhea	56 (25.8)
Dysmenorrhea: painful menstruation	154 (70.9)
Heavy bleed than normal bleed	91 (41.9)
Normal cycle lengthened	99 (45.6)
Normal cycle shortened	73 (33.6)
*Do you suffer from premenstrual symptoms? (N* = *217)*	
No	15 (7)
Yes	202 (93)
*Do you use medication to relieve menstrual irregularities? (N* = *217)*	
No	86 (39.6)
Yes	131 (60.4)
*If yes, which of the following medication do you need? (N* = *131)*	
Allopathic	22 (16.7)
Allopathic and herbal medication	16 (12.2)
Allopathic, herbal medication and home remedies	16 (12.2)
Allopathic and home remedies	5 (3.8)
Herbal medication	24 (19)
Herbal medication and home remedies	35 (26.7)
Home remedies	13 (2.2)
*Do you think that this medication is effective? (N* = *131)*	
No	23 (17.6)
Yes	108 (82.4)
*Is there a family history of menstrual irregularities? (N* = *217)*	
No	122 (56.2)
Yes	95 (43.8)
*Did you seek any medical help from any clinician?*	
No	159 (73.2)
Yes	58 (26.8)
*Did you discuss this problem with a family member or a friend?*	
No	52 (24)
Yes	165 (76)
*Did you miss classes when you had menstrual irregularities?*	
No	99 (45.6)
Sometimes	92 (42.3)
Yes	26 (12.1)

**Table 3:** Relationship between menstrual irregularities during exams and students’ demographics, academic year and age at menarche

Variable	Menstrual irregularities		χ^2^	*p*-value
	No	Yes		
	No. (%)	No. (%)		
*Age*				
18–24	223 (51.6)	209 (48.4)	0.1	0.743
25–31	10 (55.6)	8 (44.4)		
*Marital status*				
Divorced	0 (0.0)	2 (100)	5.19	0.074
Married	1 (16.7)	5 (83.3)		
Single	232 (52.5)	210 (47.5)		
*Academic year*				
2nd	58 (49.6)	59 (50.4)	2.34	0.673
3rd	36 (49.3)	37 (50.7)		
4th	73 (56.6)	56 (43.4)		
5th	34 (47.2)	38 (53.8)		
6th	32 (54.2)	27 (45.8)		
Age at menarche (mean ± SD)	12.86 ± 1.49	12.72 ± 1.61	0.77*	0.436

* = U test

A non-significant relationship was found between students’ age and type of menstrual irregularities, premenstrual symptoms, medication use, medical and family consultation, or missing classes due to menstrual irregularities (*p* > 0.05) (Table 4). A non-significant relationship was found between academic year and type of menstrual irregularities, premenstrual symptoms, medication use, medical and family consultation, or missing classes due to menstrual irregularities (*p* > 0.05) (Table 5).

**Table 4:** Relationship between students’ age and type of menstrual irregularities, premenstrual symptoms, medication use, medical and family consultation and missing classes due to menstrual irregularities

Variable	Age (years)		χ^2^	*p*-value
	18–24	25–30		
	No. (%)	No. (%)		
*Type pf menstrual irregularities (N* = *217)*				
Amenorrhea: absence of menstrual periods	84 (97.7)	2 (2.3)	0.79	0.672
Oligomenorrhea	55 (98.2)	1 (1.8)	0.81	0.665
Dysmenorrhea	147 (95.5)	7 (4.5)	1.12	0.569
Heavy bleed than normal bleed	87 (95.6)	4 (4.4)	0.31	0.855
Normal cycle lengthened	93 (93.9)	6 (6.1)	2.77	0.249
Normal cycle shortened	71 (97.3)	2 (2.7)	0.36	0.834
*Do you suffer from premenstrual symptoms? (N* = *217)*				
No	15 (100)	0 (0.0)	0.67	0.713
Yes	194 (96)	8 (4)		
*Do you use medication to relieve menstrual irregularities? (N* = *217)*				
No	83 (96.5)	3 (3.5)	0.12	0.941
Yes	126 (96.2)	5 (3.8)		
*Do you think that this medication is effective? (N* = *131)*				
No	23 (100)	0 (0.0)	1.06	0.588
Yes	103 (95.4)	5 (4.6)		
*Did you seek any medical help from any clinician?*				
No	153 (96.2)	6 (3.8)	0.11	0.942
Yes	56 (96.6)	2 (3.4)		
*Did you discuss this problem with a family member or a friend?*				
No	48 (92.3)	4 (7.7)	2.96	0.227
Yes	161 (97.6)	4 (2.4)		
*Did you miss classes when you had menstrual irregularities?*				
No	97 (98)	2 (2)	5.04	0.169
Sometimes	89 (96.7)	3 (3.3)		
Yes	23 (88.5)	3 (11.5)

**Table 5:** Relationship between students’ academic year and type of menstrual irregularities, premenstrual symptoms, medication use, medical and family consultation and missing classes due to menstrual irregularities

Variable	Academic year		χ^2^	*p*-value
	Basic years (2nd, 3rd) No. (%)	Clinical years (4th, 5th, 6th) No. (%)		
*Type pf menstrual irregularities (N* = *217)*				
Amenorrhea	42 (48.8)	44 (51.2)	1.93	0.38
Oligomenorrhea	26 (46.4)	30 (53.6)	0.84	0.655
Dysmenorrhea: painful menstruation	66 (42.9)	88 (57.1)	1.11	0.573
Heavy bleed than normal bleed	43 (47.3)	48 (52.7)	1.28	0.527
Normal cycle lengthened	51 (51.5)	48 (48.5)	4.65	0.098
Normal cycle shortened	38 (52.1)	35 (47.9)	3.45	0.178
*Do you suffer from premenstrual symptoms? (N* = *217)*				
No	7 (46.7)	8 (53.3)	0.73	0.691
Yes	89 (44.1)	113 (55.9)		
*Do you use medication to relieve menstrual irregularities? (N* = *217)*				
No	37 (43)	49 (57)	0.78	0.675
Yes	59 (45)	72 (55)		
*Do you think that this medication is effective? (N* = *131)*				
No	9 (39.1)	14 (60.9)	1.25	0.533
Yes	51 (46.8)	57 (53.2)		
*Did you seek any medical help from any clinician?*				
No	75 (47.2)	84 (52.8)	2.79	0.247
Yes	21 (36.2)	37 (63.8)		
*Did you discuss this problem with a family member or a friend?*				
No	20 (38.5)	32 (61.5)	1.63	0.442
Yes	76 (46.1)	89 (53.9)		
*Did you miss classes when you had menstrual irregularities?*				
No	45 (45.5)	54 (54.5)	0.82	0.844
Sometimes	40 (43.5)	52 (56.5)		
Yes	11 (42.3)	15 (57.7)		

Oligomenorrhea, a heavier than normal bleed, a longer normal cycle, using medications to relieve pain and missing classes due to menstrual irregularities were significantly higher among single students (*p* < 0.05) (Table 6). However, a non-significant relationship was found between students’ marital status and amenorrhea, dysmenorrhea, shortening of the normal cycle, premenstrual symptoms, medication use, and medical or family consultations (*p* > 0.05).

**Table 6:** Relationship between students’ marital status and type of menstrual irregularities, premenstrual symptoms, medication use, medical and family consultation and missing classes due to menstrual irregularities

Variable	Marital status			χ^2^	*p*-value
	Divorced No. (%)	Married No. (%)	Single No. (%)		
*Type of menstrual irregularities (N* = *217)*					
Amenorrhea	0 (0.0)	2 (2.3)	84 (97.7)	7.93	0.094
Dysmenorrhea	2 (1.3)	3 (1.9)	149 (96.8)	7.4	0.116
Normal cycle shortened	1 (1.4)	3 (4.1)	96 (94.5)	8.44	0.077
*Type pf menstrual irregularities (N* = *217)*					
Oligomenorrhea					
No	2 (1.2)	5 (3.1)	154 (95.7)	9.72	**0.045**
Yes	0 (0.0)	0 (0.0)	56 (100)		
No Heavy bleed than normal bleed					
No	0 (0.0)	4 (3.2)	122 (96.8)	12.65	**0.013**
Yes	2 (2.2)	1 (1.1)	88 (96.7)		
No Normal cycle lengthened					
No	0 (0.0)	2 (1.7)	116 (98.3)	10.92	**0.027**
Yes	2 (2)	3 (3)	94 (94.9)		
*Do you suffer from premenstrual symptoms? (N* = *217)*					
No	0 (0.0)	1 (6.7)	14 (93.3)	7.82	0.098
Yes	2 (1)	4 (2)	196 (97)		
*Do you use medication to relieve menstrual irregularities? (N* = *217)*					
No	0 (0.0)	4 (4.7)	82 (95.3)	13.83	0.008
Yes	2 (1.5)	1 (0.8)	128 (97.7)		
*Do you think that this medication is effective? (N* = *131)*					
No	0 (0.0)	0 (0.0)	23 (100)	6.86	0.143
Yes	2 (1.8)	1 (0.9)	105 (97.2)		
*Did you seek any medical help from any clinician?*					
No	2 (1.3)	3 (1.9)	154 (96.9)	7.49	0.112
Yes	0 (0.0)	2 (3.4)	56 (96.6)		
*Did you discuss this problem with a family member or a friend?*					
No	0 (0.0)	1 (1.9)	51 (98.1)	6.59	0.159
Yes	2 (1.2)	4 (2.4)	159 (96.4)		
*Did you miss classes when you had menstrual irregularities?*					
No	1 (1)	5 (5.1)	93 (93.9)	16.22	**0.013**
Sometimes	1 (1.1)	0 (0.0)	91 (98.9)		
Yes	0 (0.0)	0 (0.0)	26 (100)		

Table 7 shows that on doing the multivariate logistic regression analysis to assess the risk factors of menstrual irregularities, none of the independent variables had a significant relationship (*p* ≥ 0.05).

**Table 7:** Multivariate logistic regression analysis of risk factors of menstrual irregularities

	B	Wald	*p*-value	Odds Ratio (CI: 95%)
Age	0.31	0.56	0.534	0.01 (0.13–1.02)
Marital status	0.5	0.61	0.132	0.2 (0.6–0.13)
Academic year	0.35	0.81	0.142	0.3 (0.3–1.09)
Age at menarche (mean ± SD)	0.9	0.86	0.073	0.98 (1.5–1.09)

## Discussion

Excessive stress during exams is one of the most common concerns among university students around the world. It can lead to physiological, hormonal, immunological, psychological, and behavioral changes, which can lead to menstrual cycle irregularity [31]. The purpose of our study was to evaluate menstrual cycle irregularities at the time of exams among female medical students at KAU in Jeddah, Saudi Arabia.

Most of the sample were single young women with a mean age of menarche at 12.79. Almost half the sample presented with menstrual cycle irregularity. Medical students are at increased risk of menstrual disorders due to lifestyles that include lack of sleep, irregular diet, and exercise habits [32]. A previous study in Riyadh, Saudi Arabia, among different university colleges found that four out of 10 female students suffered from menstrual cycle disturbance [31]. This could be the result of psychological stress, which may lead to an imbalance between estrogen and progesterone ratios [31–33]. In contrast, another study in India found that the level of stress among medical students was above average, or the stress level was higher compared to non-medical students, but this stress did not, in general, affect the regularity of the women’s periods among their sample [33].

The most prevalent menstrual irregularities reported by the students in the current study were dysmenorrhea, followed by cycle lengthening, and heavy bleeding. These results support a study done in Saudi Arabia that found that dysmenorrhea was the most common irregularity [34]. The majority of participants in this study had dysmenorrhea during the exam period, and this supports findings by Kollipaka et al. which found that dysmenorrhea was the most common menstrual abnormalities reported by students [35]. A study in Nigeria also found that dysmenorrhea was the most common problem associated with female medical students [36].

The current study showed that most students with any form of irregularity can be assisted with medication, especially herbal medication and home remedies, and this reduced the number of classes skipped due to menstrual irregularity. A study done in 2018 also found that herbal medication can play a significant role in treating menstrual irregularity [37].

Several studies discuss the relationship between the academic level and menstrual cycle irregularity. In this study, a non-significant relationship indicated that most students with menstrual changes were in the first 3 years of study. On the other hand, a study done in Al-Ahsa, Saudi Arabia, found that students in clinical years had high levels of stress and were two times more likely to suffer from menstrual changes [34]. A study in Dammam, Saudi Arabia, found that many students exposed to similar levels of stress had normal menstruation [21]. However, the current study suggests that there is a high prevalence of dysmenorrhea among medical students, most of whom were in the clinical years. Previous literature showed that the most frequent gynecological condition among female medical students is dysmenorrhea [20, 38]. In contrast, a study by Ibrahim et al. found that the prevalence of dysmenorrhea was considerably greater among students in the basic years which is the first 3 years in medical school compared to those in the clinical years which Is the last 3 years [22]. This may be due to the pressure of adapting to a new location, which may put a psychological burden on medical students in the clinical years who have recently entered hospital settings.

In addition, the menstrual cycle lengthened in almost half of students in this study. In contrast, a study in Dammam, Saudi Arabia, found that the majority of participants had a normal length cycle [21]. The current study showed that more than one-third of participants reported that their periods were heavier than usual, compared with a cross‐sectional study done among medical students that found almost two-thirds of students reported normal blood loss [21].

In this study, almost half the students had premenstrual symptoms. A study in Jeddah, Saudi Arabia, reported a strong positive relationship between both premenstrual symptoms and stress levels [39]. A study in South-Eastern Nigeria found that less than half of students had premenstrual symptoms [40]. Also, more than half the students used medication to relieve the pain, similar to a study in India that found that minorities use painkillers to relieve menstrual pain [33].

The current study found no significant relationship between the age of the students and type of menstrual irregularities, similar to a study in South-Eastern Nigeria that found no relationship between age and menstrual changes (*p* = 0.901). This may be because the ages were very close to each other [40].

## Limitations

This study has several limitations, despite using a validated questionnaire with a large sample size. A limitation was that it was a cross-sectional study. The study was limited to students in one university, which may not represent the general population of women. The menstrual patterns were based on students’ self-reports, rather than clinical examinations or hormone measurements, which may have led to skewing of the results. Some factors that may be confounders were not assessed in this study such as the usual menstrual pattern of the participant, use of oral contraceptive pills, and other habits during the exams period that may have effects on the normal cycle such as sleep deprivation, caffeine intake, physical activity, quality of food, smoking, and level of psychological stress. Future research could be conducted in other colleges and include questions about these factors.

## Recommendations

This study showed that female medical students have a significant frequency of menstruation abnormalities during exams. Based on these results, and to minimize the negative effects of the educational process, it is recommended that universities educate female students about relaxation techniques, such as yoga, before the exams. Also, females should have a better understanding of menstrual pain and perceptions of treatment choices could be improved.

## Conclusion

Our study aimed to detect menstrual cycle irregularities during exam among female medical students in King Abdulaziz University. According to that, we find significant frequency of menstruation abnormalities during exams. Which indicate that exam can disturb the menstrual cycle. Consequently, we recommend universities to educate female students about relaxation techniques, such as yoga. Also, they should have a better understanding of menstrual pain and their treatment choices.

## Data Availability

The corresponding author can provide access to the datasets generated and analyzed during the current investigation based on request at any time.
